# A Synchrony-Dependent Influence of Sounds on Activity in Visual Cortex Measured Using Functional Near-Infrared Spectroscopy (fNIRS)

**DOI:** 10.1371/journal.pone.0122862

**Published:** 2015-03-31

**Authors:** Ian M. Wiggins, Douglas E. H. Hartley

**Affiliations:** 1 National Institute for Health Research (NIHR) Nottingham Hearing Biomedical Research Unit, Nottingham, United Kingdom; 2 Otology and Hearing Group, Division of Clinical Neuroscience, School of Medicine, University of Nottingham, Nottingham, United Kingdom; 3 Medical Research Council (MRC) Institute of Hearing Research, Nottingham, United Kingdom; CEA.DSV.I2BM.NeuroSpin, FRANCE

## Abstract

Evidence from human neuroimaging and animal electrophysiological studies suggests that signals from different sensory modalities interact early in cortical processing, including in primary sensory cortices. The present study aimed to test whether functional near-infrared spectroscopy (fNIRS), an emerging, non-invasive neuroimaging technique, is capable of measuring such multisensory interactions. Specifically, we tested for a modulatory influence of sounds on activity in visual cortex, while varying the temporal synchrony between trains of transient auditory and visual events. Related fMRI studies have consistently reported enhanced activation in response to synchronous compared to asynchronous audiovisual stimulation. Unexpectedly, we found that synchronous sounds significantly reduced the fNIRS response from visual cortex, compared both to asynchronous sounds and to a visual-only baseline. It is possible that this suppressive effect of synchronous sounds reflects the use of an efficacious visual stimulus, chosen for consistency with previous fNIRS studies. Discrepant results may also be explained by differences between studies in how attention was deployed to the auditory and visual modalities. The presence and relative timing of sounds did not significantly affect performance in a simultaneously conducted behavioral task, although the data were suggestive of a positive relationship between the strength of the fNIRS response from visual cortex and the accuracy of visual target detection. Overall, the present findings indicate that fNIRS is capable of measuring multisensory cortical interactions. In multisensory research, fNIRS can offer complementary information to the more established neuroimaging modalities, and may prove advantageous for testing in naturalistic environments and with infant and clinical populations.

## Introduction

Perceptual judgments often reflect a combination of inputs from multiple senses [1]. The neural mechanisms that support this combining of information across the sensory modalities are not fully understood. Over recent years, converging evidence from human neuroimaging studies and single-unit recordings in animals has revealed that interactions among the senses arise early in cortical processing, including in primary sensory cortices, which were traditionally considered to be strictly unisensory (for reviews see [2–5]). Our focus here is on the human neuroimaging results. Whilst the most appropriate statistical criterion for identifying multisensory interactions in neuroimaging data is a subject of ongoing debate [6–8], it is widely accepted that multisensory effects can modulate both the amplitude [9, 10] and temporal dynamics [11] of the blood oxygenation level-dependent (BOLD) fMRI signal in primary sensory cortices. Other studies have exploited the temporal resolution of electroencephalography (EEG) and magnetoencephalography (MEG) to confirm that multisensory interactions occur early in cortical processing [12–15], and, more recently, to study the putative role of synchronized oscillatory brain activity in mediating these effects [16, 17].

In the present study, we investigated whether functional near-infrared spectroscopy (fNIRS) is capable of measuring multisensory interactions in what was traditionally considered ‘sensory-specific’ cortex. As a relatively quiet, non-invasive, and low-cost technique, fNIRS continues to gain popularity as a neuroimaging tool with a number of practical advantages [18, 19]. Although a recent fNIRS study reported multisensory interactions in 3-month-old infants [20], to our knowledge this is the first application of fNIRS to the study of multisensory processing in adults. Specifically, we investigated a modulatory influence of sounds on activity in visual cortex. Visual cortex, located in the occipital lobe, is relatively accessible using fNIRS [21], and has been the target of several fNIRS studies that used unisensory visual stimulation [22–28]. Furthermore, robust auditory influences on activity in visual cortex have been well documented, both in animal electrophysiological studies [29–31], and using a variety of imaging techniques in humans [10–12, 14, 32–34]. It has been suggested that the primary role of such auditory influences in non-auditory cortices may be to modulate response gain within the relevant modality, in this case vision [35].

Temporal, spatial, and semantic congruency are known to be key factors in determining whether the brain integrates multimodal sensory inputs [36, 37]. We studied temporal congruency, minimizing semantic and spatial influences through the use of simple, stationary stimuli. Specifically, we investigated the role of temporal synchrony between trains of transient auditory and visual events, following the example of several previous fMRI studies [9, 38–40]. A consistent finding across these fMRI studies was that synchronous audiovisual stimulation generally enhanced the strength of stimulus-related cortical activations, while asynchronous stimulation generally had a suppressive effect. This pattern of enhancement and suppression was observed not only in established multisensory areas (e.g., superior temporal sulcus, STS), but also in primary auditory and visual cortices, using a variety of experimental paradigms. Enhancement of cortical responses to synchronous versus asynchronous audiovisual stimulation was typically also associated with improved behavioral performance, even when task-relevant information was provided in only one of the stimulated modalities [39, 40].

In the present study, we adapted the fMRI paradigms employed by Noesselt et al. [9] and Marchant et al. [40] to suit the fNIRS imaging modality. Specifically, we used visual stimuli that have been shown in previous studies to produce robust fNIRS responses from visual cortex [24, 25]. We measured visual cortical responses using fNIRS while participants were presented with trains of unpredictably timed, transient auditory and visual events that were either synchronous or asynchronous. We also measured responses to matching unisensory baseline conditions. Following Marchant et al. [40], we simultaneously measured behavioral performance in detecting occasional higher-intensity targets. In short, we aimed to measure a synchrony-dependent influence of sounds on activity in visual cortex using the emerging brain-imaging technique fNIRS. Based on the aforementioned fMRI studies, we anticipated enhancement of responses by synchronous sounds and suppression by asynchronous sounds.

## Materials and Methods

### Overview

**Fig 1:**
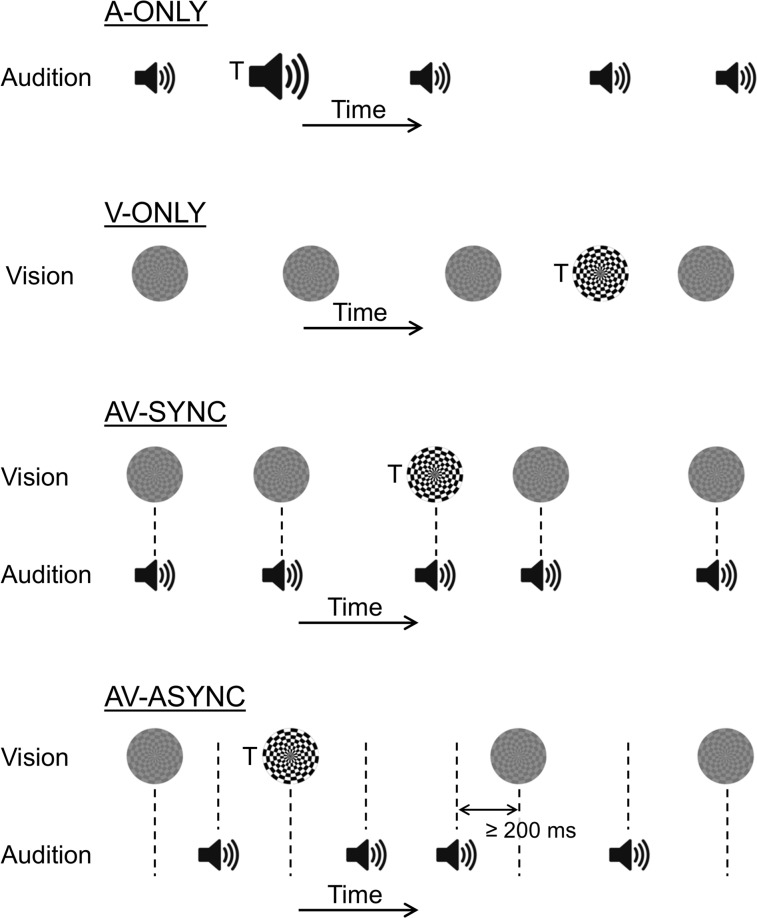
Schematic Representation of Stimulus Timing in Each Stimulation Condition. The exact timing of events was in all cases arrhythmic and unpredictable. Events marked with a “T” represent higher-intensity (A-ONLY condition) or higher-contrast (V-ONLY, AV-SYNC, and AV-ASYNC) targets, which occurred every 2–5 s. In the AV-ASYNC condition, onsets between adjacent auditory and visual events were separated by a minimum of 200 ms.

Functional NIRS measurements were collected simultaneously while participants performed a behavioral target-detection task. Trains of transient auditory and/or visual events with unpredictable, arrhythmic timing were presented in four stimulation conditions (Fig 1): auditory-only (A-ONLY), visual-only (V-ONLY), audiovisual with synchronous events (AV-SYNC), and audiovisual with asynchronous events (AV-ASYNC). These four conditions were each presented five times in random order in a block design. Our motivation for using a block design was twofold: Firstly, following previous fMRI studies [9, 38–40], we aimed to assess the influence of temporal synchrony on the aggregate response to trains of auditory and visual events, rather than the response to isolated individual events; secondly, given that fNIRS generally has significantly poorer signal-to-noise ratio than fMRI [41, 42], we wished to maximize our ability to measure robust cortical responses, which is facilitated by the high efficiency of a block design [43]. Stimulation blocks were 20 s in duration and the intervening rest periods had random durations in the range 20–40 s. The total measurement time was approximately 17 minutes. Participants were given the opportunity to practice the behavioral task before measurements began, experiencing two repetitions of each stimulation condition in random order. Testing took place in a double-walled sound booth with dimmed lighting.

### Participants and ethical approval

Twenty-four participants (mean age 27 years, 9 males) with no history of any neurological disorder took part in the study after giving written informed consent. All participants had normal hearing (< = 25 dB HL at audiometric test frequencies between 500 Hz and 4 kHz) and normal or corrected-to-normal vision. Most participants (*N* = 21) were right-handed as assessed using the Edinburgh Handedness Inventory [44]. The study was approved by the Nottingham 1 Research Ethics Committee.

### Stimulus timing

Care was taken to ensure that the temporal statistics of the event sequences were closely matched across all stimulation conditions. In the AV-SYNC condition, each visual event was accompanied by an auditory event with a synchronous onset. In the AV-ASYNC condition, the timings of visual and auditory events were derived independently, with an additional constraint that all visual event onsets were separated from the nearest auditory event onset by at least 200 ms. Stimulus timing was confirmed by measurement using a response-time box that provided inputs for a photodiode and a direct audio connection (RTBox [45]).

The precise method used to generate the event timings was as follows: First, a random sequence of visual event times was generated, in which the stimulus onset asynchrony (SOA) between successive events was between 100 and 500 ms (randomly selected from a uniform distribution). The event times were then quantized so as to coincide exactly with the refresh rate (60 Hz) of the visual display unit (VDU), to ensure precise timing of visual stimuli. A sequence of auditory event times was generated in a similar manner, but without quantization to the VDU refresh rate. The following procedure was then used to remove any cases in which, by chance, visual and auditory events fell within 200 ms of one another. The visual event times were stepped through one by one, and for each visual event the nearest auditory event was determined. If the SOA between these was less than 200 ms, then either the visual event or the auditory event was removed from the corresponding sequence with equal probability. The scan was then restarted from the first remaining visual event. This procedure was repeated until no visual and auditory events had onsets within 200 ms of one another. In the resulting sequences, events occurred at a mean rate of 1.68 Hz (*SD* = 0.25) in each modality. To enforce synchronicity in the AV-SYNC condition, the sequence of auditory event times was set equal to the sequence of visual event times. In the AV-ASYNC condition, we did not actively constrain the number of consecutive events that could occur in one modality before the next event in the other modality.

### Behavioral task

Following Marchant et al. [40], we used a suprathreshold target-detection task, in which participants responded as quickly as possible to occasional “target” events presented within a train of “standard” events. Randomly selected events from the pre-calculated sequences were designated as targets, with a target occurring every 2–5 s. In total, each participant was presented with an average of 19.7 (*SD* = 1.59) targets in each stimulation condition, with no systematic difference in the number of targets between conditions. Because our fNIRS measurements targeted visual cortex, we focused on the visual domain in the behavioral task also. Thus, in all conditions that included visual stimulation (V-ONLY, AV-SYNC, and AV-ASYNC), the task was to identify occasional higher-contrast visual targets. Participants were instructed to press a button as quickly as possible whenever they saw a higher-contrast checkerboard, while maintaining accuracy. In the same conditions, all auditory events were presented at an identical sound pressure level, and so provided no task-relevant information. Participants were advised that they could ignore any sounds that happened to be presented alongside the visual stimuli. To ensure that participants remained attentive and to control for any effect of motor responses, a control task was implemented in the A-ONLY condition: auditory targets were distinguished from the standard events by an increase in sound pressure level, and participants were instructed to respond as quickly as possible whenever they heard a louder sound.

Prior to commencing the main task, the increase in contrast ratio that differentiated visual targets from standard events (V-ONLY, AV-SYNC, and AV-ASYNC conditions), and the increase in sound pressure level that served an equivalent role in the A-ONLY condition, were set on an individual basis to ensure approximately 70% target-detection accuracy. Individual thresholds for these target intensity increments were determined under unimodal conditions using a two-down, one-up adaptive staircase procedure, first for the auditory modality and then for vision. A correct response was defined as a button press occurring within a 1-s period following presentation of a target; a button press at any other time was interpreted as an incorrect response, as was a missed target. Before each adaptive procedure began, participants were given a few minutes practice in a condition in which the target events could easily be distinguished from the standard events.

Responses were collected using a dedicated button box that contained its own microprocessor and high-resolution clock to ensure accurate timing (RTBox [45]). Behavioral performance on the main task was quantified by accuracy and response-time measures. A button press occurring between 150 and 1000 ms after the onset of a target was considered a hit. Button presses at other times, including multiple presses following a single target, were classified as false alarms. A target which was not followed by a button press within the valid time window was classified as a miss. Response time was assessed for hits only. Similarly to Johnson and Zatorre (46], we calculated accuracy using the formula: 100 × [(hits − false alarms) ÷ (hits + misses)].

### Visual stimulation

Each visual event was a transiently presented polar checkerboard (nominal duration 33.3 ms). The checkerboard contrast was reversed between successive presentations. The centrally located checkerboards subtended a visual angle of 16° and were divided into 8 rings and 24 wedges. The light and dark elements of the standard checkerboards had luminance 69 cd/m^2^ and 51 cd/m^2^, respectively (measured with a MAVO-SPOT 2 USB meter, Gossen, Germany), giving a Michelson contrast of 15%. The target stimuli in the V-ONLY, AV-SYNC, and AV-ASYNC conditions had a higher contrast achieved by simultaneously increasing the luminance of the light elements and decreasing the luminance of the dark elements, according to the prior measurement of individual target-detection sensitivity. The checkerboards were presented against a uniform gray background (58 cd/m^2^). A central white cross subtending a visual angle of 1° was presented continuously throughout the experiment. Participants were instructed to maintain fixation on this central cross at all times. Visual stimuli were presented on a 22” liquid crystal display viewed from a distance of 75 cm.

Note that our use of centrally located visual stimuli covering a relatively large proportion of the visual field was at odds with the studies of Noesselt et al. [9] and Marchant et al. [40], in which smaller, peripherally presented (~8–18° eccentricity) stimuli were used (simple colored shapes and rectangular checkerboards, respectively). While numerous studies have demonstrated that multisensory enhancement can occur for centrally presented stimuli [38, 47–50], it has been suggested that the use of peripheral visual stimuli might maximize the opportunity for interaction between the auditory and visual modalities [51]. These interactions could be mediated by direct cortico-cortical connections between early auditory and visual cortices, which neuroanatomical studies in non-human primates have found to terminate predominantly in areas that represent the peripheral visual field [52, 53]. In the present study, we used larger, centrally located visual stimuli based on previous fNIRS studies that reported robust responses from visual cortex [24, 25]. Pilot testing conducted in our laboratory suggested that we could not measure robust responses to peripheral visual stimuli, possibly owing to the limited depth penetration of fNIRS [41], combined with the fact that the peripheral visual field maps to anterior areas of visual cortex that are further from the surface of the head [54]. Indeed, measuring responses to stimulation in the peripheral visual field using optical imaging can be challenging, even using a state-of-the-art high-density diffuse optical tomography system [27].

### Auditory stimulation

Each auditory event was a brief (10-ms duration including 1-ms linear ramps) 1-kHz tone-pip. The level of the standard events was 76 dB SPL. The level of the target stimuli in the A-ONLY condition was increased from this baseline according to the prior measurement of individual target-detection sensitivity. Auditory stimuli were presented from a pair of small loudspeakers positioned on either side of, and vertically centered with, the VDU. Site constraints meant that it was not possible to house the fNIRS equipment outside the sound booth during testing, although a dense sound-absorbing screen was placed between the equipment and the area in which participants were seated. The steady ambient noise level at the participant’s position was 38 dB SPL (A-weighted), dominated by low-to-mid-frequency fan noise from the fNIRS equipment.

### fNIRS measurements and analyses

Measurements were made using a continuous-wave fNIRS system (ETG-4000, Hitachi Medical Co., Japan). A 3 x 5 optode array (comprising 8 emitters and 7 detectors) was used, giving 22 measurement channels in total. The inter-optode spacing was 30 mm. The ETG-4000 measures simultaneously at wavelengths of 695 nm and 830 nm (sampling rate 10 Hz), and uses frequency modulation to minimize crosstalk between wavelengths and optodes. For a comprehensive review of the principles and practicalities of continuous-wave fNIRS, see [55].

**Fig 2:**
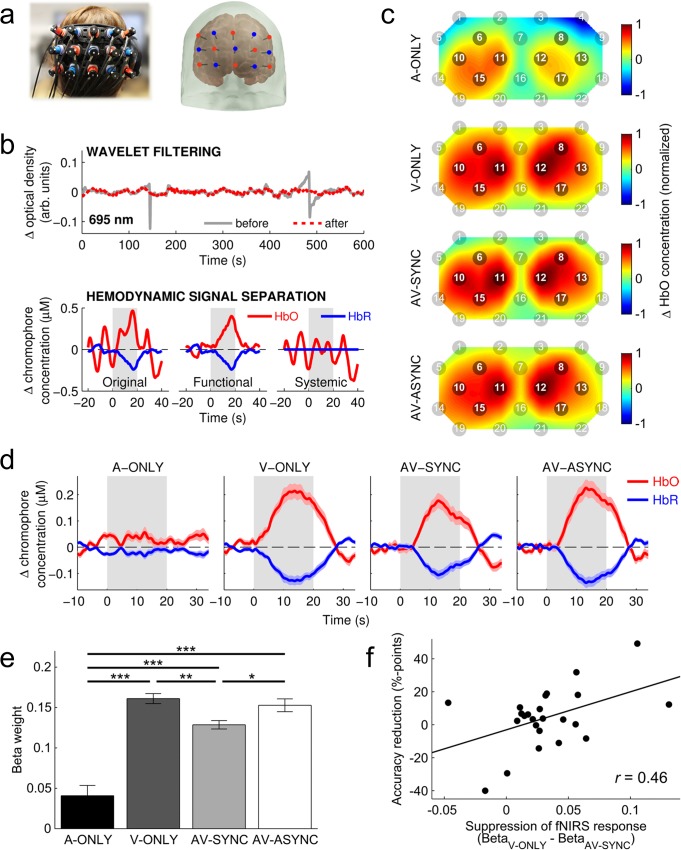
fNIRS Analysis and Results. (a) Photograph of the optode array on one participant and corresponding optode positions registered to an atlas brain. (b) Representative examples of the effect of two key signal processing stages: (upper panel) wavelet filtering successfully removes spikes from the optical signal, while leaving portions of the signal that are not contaminated by motion artifacts unchanged; (lower panel) for a hemodynamic response function showing substantial contamination by physiological noise, the hemodynamic signal separation technique successfully recovers a plausible functional response, plus an estimate of the systemic interference. (c) Group-average patterns of activation (normalized mean change in HbO between 6 and 20 s post onset) across the array for each stimulation condition. Channels within the predefined ROI (Ch# 6, 8, 10–13, 15, and 17) are highlighted. (d) Group-average hemodynamic response functions within the ROI for each stimulation condition. The shaded gray area shows the stimulation period. (e) Mean beta weights from the GLM analysis. Error bars show ±1 standard error, corrected to account for the repeated-measures nature of the design [57]. Asterisks denote a statistically significant difference between the conditions indicated (* *p* <. 05; ** *p* <. 01; *** *p* <. 001; Bonferroni corrected). (f) Assessment of a possible relationship between the suppressive effect of synchronous sounds on the fNIRS response from visual cortex (Beta_V-ONLY_—Beta_AV-SYNC_) and a corresponding reduction in the accuracy of visual target detection (Accuracy_V-ONLY_ − Accuracy_AV-SYNC_). While Pearson’s correlation suggested a significant linear relationship (*r* = .46, *p* = .023, two-tailed), this result could not be confirmed by a robust regression analysis (*p* = .136).

The optode array was initially placed over the occipital lobe with the center optode aligned roughly over position Oz of the international 10–20 coordinate system [56]. To ensure (as far as possible) that the measurement channels were positioned consistently across individuals, we followed the approach of Plichta et al. [25] and conducted a short functional localizer experiment. This comprised three cycles of high-contrast checkerboard stimulation (96% Michelson contrast, 16-Hz reversal rate, 10-s stimulation blocks, 20-s rest periods). The resulting activation pattern was viewed as a topographic 2D map using the ETG-4000’s built-in analysis software. If necessary, the position of the array was adjusted and the localizer experiment repeated until the activation pattern was centered and symmetrical. Fig 2A shows the final optode positions on one participant, who provided written informed consent for publication of this image. Once the position of the array was finalized, an elastic cotton bandage was gently wrapped around the participant’s head to help maintain secure contact between the optodes and the scalp. Participants were asked to sit as still as possible to reduce motion artifacts, although for comfort no head restraint was used.

Analysis of the fNIRS data was performed in MATLAB (MathWorks, Natick, MA). First, we aimed to exclude any “bad channels” that were clearly influenced by unstable or weak optode contact. Guided by Umeyama and Yamada [58], we identified channels suffering from “unstable” optode contact by unusually high variance in the signal baseline (low-pass filtered at 0.1 Hz), and channels suffering from “weak” optode contact by unusually high variance due to high-frequency noise (high-pass filtered at 1.0 Hz). We calculated the long-term variance in these two frequency regions and assessed each measurement in relation to the pooled distribution across both wavelengths and all participants. By visual inspection of the distributions, we set as a threshold for exclusion a variance further than one standard deviation from the mean. A channel was excluded if this threshold was exceeded at either wavelength and in either frequency region. For 19 of the 24 participants, no channels were excluded. For the remaining five participants, between 4 and 12 channels (out of a total of 22) were excluded. The excluded channels generally corresponded to ones that had been noted as problematic at the time of testing, usually because of issues with hair obscuring good optode contact. We separately confirmed that excluding these five participants outright, instead of excluding only the problematic channels, did not alter the conclusions reported in this manuscript.

After excluding bad channels, the raw intensity signals were converted to changes in optical density [59]. We then applied wavelet filtering to correct for motion artifacts. Motion artifacts are frequently encountered in fNIRS data, usually resulting from differential movement between the optical fibres and the scalp, and typically manifesting as abrupt spikes or jumps in intensity in the optical signal. Wavelet filtering has emerged in recent years as a promising approach to correcting for these artifacts [60, 61]. We used the *hmrMotionCorrectWavelet* function included in the HOMER2 fNIRS processing package [59], which performs a simplified version of the algorithm described by Molavi and Dumont [62]. The algorithm applies a probability threshold to remove outlying wavelet coefficients, which are assumed to correspond to motion artifacts. We used a threshold of 1.219 times the inter-quartile range, equivalent (assuming a Gaussian distribution of wavelet coefficients) to the α = 0.1 threshold adopted in past studies [60, 61]. Fig 2B shows a representative example of how this approach removed motion artifacts without affecting uncontaminated portions of the signal.

Following motion-artifact correction, the optical density signals were band-pass filtered between 0.01 and 0.5 Hz to attenuate low-frequency drift and cardiac oscillations, and then converted into estimates of changes in the concentration of oxygenated (HbO) and de-oxygenated (HbR) hemoglobin using the modified Beer-Lambert law [63]. We used a default value of 6 for the differential path-length factor at both wavelengths, noting that this may diminish the accuracy of the estimated absolute concentration changes because it does not account for the partial volume effect associated with focal cortical activation [64]. Because band-pass filtering is only partially effective in removing physiological noise from fNIRS measurements [59], we additionally employed the hemodynamic signal separation method described by Yamada et al. [65]. This algorithm separates the hemodynamic signal into estimated functional and systemic components based on the assumption that changes in HbO and HbR will be negatively correlated in the functional component, but positively correlated in the systemic component (see Fig 2B for a representative example). Only the functional component was retained for further analysis.

We defined an *a priori* region of interest (ROI) based on the results of past studies that used the same fNIRS system, similar visual stimuli, and similar optode-array placement [24, 25]. The ROI comprised eight measurement channels (four on the left side and four on the right) covering the areas in which we expected to observe visual activation (see Fig 2C). We did not define a non-ROI, since our 3 x 5 array did not offer any measurement channels that were sufficiently far from the expected areas of activation. The more distant non-ROI channels of the 3 x 11 optode array used by Plichta et al. [24, 25] were not available in our setup. While the lack of an anatomical image and limited spatial resolution of fNIRS preclude the accurate localization of responses to specific sub-areas of visual cortex, the available evidence from combined fNIRS–fMRI studies [42, 66] suggests that our measurements most probably sampled the superficial portion of primary visual cortex (area V1) and the surrounding extrastriate cortex (areas V2/V3).

For visualizing hemodynamic responses (Fig 2C and 2D), the time course of HbO and HbR concentration changes in each measurement channel was block-averaged using the HOMER2 *hmrBlockAvg* function [59]. We used a general linear model (GLM) to quantify the strength of the response to each stimulation condition. The design matrix included four boxcar regressors (one for each condition), which were convolved with the canonical hemodynamic response function provided in SPM8 [http://www.fil.ion.ucl.ac.uk/spm]. Beta weights were extracted and averaged across the measurement channels included in the predefined ROI, before being subjected to statistical analysis. We quantified the magnitude of the fNIRS response across the ROI as a whole, rather than on a channel-by-channel basis, as we did not anticipate substantial spatial variation amongst the channels included in the ROI; nor did we predict any substantial difference between left and right hemispheres. Preliminary analysis of our data (not shown) supported these predictions. Although we present results primarily in terms of the HbO parameter for simplicity, because the hemodynamic signal separation method assumes a fixed linear relationship between HbO and HbR in the functional response, the results of all statistical analyses were identical regardless of whether conducted on the HbO or HbR data.

## Results

### Activation of visual cortex measured using fNIRS

We first assessed the spatial distribution of activation across the optode array for each stimulation condition to confirm the suitability of the predefined ROI (Fig 2C). Group-average activation patterns were derived from the mean change in HbO concentration from 6 to 20 s post stimulation-block onset. The activation patterns were normalized independently for each condition to better illustrate the spatial distribution of activation, irrespective of overall response strength. The activation pattern was similar in the three conditions that included visual stimulation (V-ONLY, AV-SYNC, and AV-ASYNC). The pattern exhibited two clear lobes, one to the left and the other to the right of midline, which aligned well with the predefined ROI. Activation in channels outside the ROI was markedly weaker, though not qualitatively different from activation within the ROI, presumably because of the close spatial proximity of all channels to the ROI, the limited spatial resolution of fNIRS, and differences in optode-array placement relative to underlying anatomy between individuals. In the A-ONLY condition, there was some evidence of positive activation in the ROI, while an isolated channel located in the top-right corner of the array (Ch# 4) appeared to show deactivation compared to baseline. Overall, these results satisfied us that the predefined ROI was appropriately located in order to calculate summary measures of visual-cortex activation by averaging across the included channels.

To confirm that plausible hemodynamic responses had been obtained, we plotted the group-average time course of concentration changes in HbO and HbR within the ROI for each stimulation condition (Fig 2D). The conditions that included visual stimulation clearly exhibited the archetypal pattern of a stimulus-locked increase in HbO and a corresponding decrease in HbR, with sluggish dynamics characteristic of neurovascular coupling. The response peaked approximately 13 s into the stimulation block, with no significant difference in peak response latency between these three conditions (Repeated-measures ANOVA, *F*(2, 46) = 0.20, *p* = .823). During the return to baseline after cessation of stimulation, the HbO/HbR traces exhibited a small under/overshoot, reminiscent of the BOLD post-stimulus undershoot commonly reported in fMRI studies [67]. The group-average response in the A-ONLY condition was much weaker, and did not obviously follow the shape of a canonical hemodynamic response.

We used the GLM beta weights to quantify differences in fNIRS response strength between stimulation conditions (Fig 2E). A repeated-measures ANOVA, with Greenhouse-Geisser correction for non-sphericity, confirmed a significant effect of condition (*F*(1.64, 37.66) = 31.50, *p* <. 001). As expected, the fNIRS response from visual cortex was significantly stronger in all conditions that included visual stimulation, compared to the auditory-only condition (all *p* <. 001, Bonferroni-corrected pairwise comparisons). Nonetheless, a single-sample *t*-test on the A-ONLY beta weights indicated that the auditory-only condition did result in significant activation in the visual ROI compared to rest (*t*(23) = 3.26, *p* = .003). Based on the findings of related fMRI studies [9, 38–40], our *a priori* hypothesis was that, compared to visual-only stimulation, activity in visual cortex would be enhanced by synchronous sounds and suppressed by asynchronous sounds. The data did not support this prediction. The fNIRS response from visual cortex was, in fact, weaker in the AV-SYNC condition than in both the V-ONLY (*p* = .001) and AV-ASYNC (*p* = .030) conditions (Bonferroni-corrected pairwise comparisons). That is, the presence of synchronous sounds led to a suppression of the fNIRS response from visual cortex. In contrast, the presence of asynchronous sounds had little effect (AV-ASYNC versus V-ONLY, n.s., *p* = .439 uncorrected).

### Behavioral performance and its relationship with fNIRS response strength

**Fig 3:**
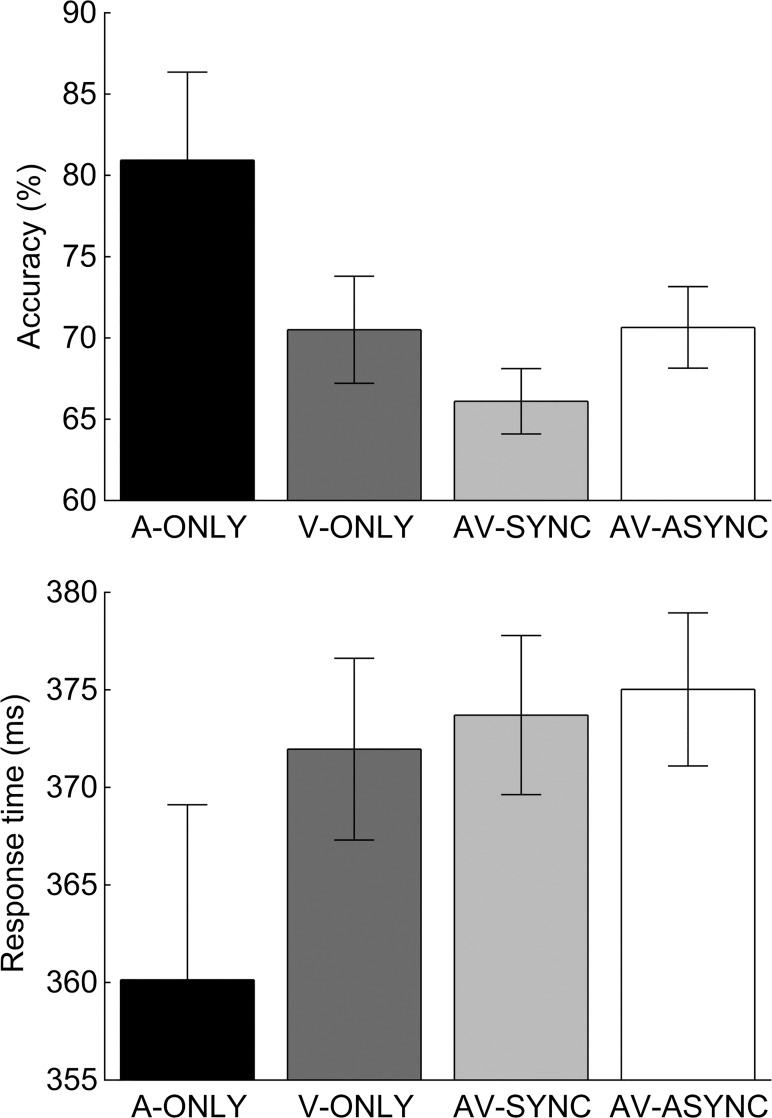
Behavioral Performance. Mean accuracy (upper panel) and response time (lower panel) for detecting occasional higher-intensity (A-ONLY condition) or higher-contrast (V-ONLY, AV-SYNC, and AV-ASYNC conditions) targets embedded within a train of standard events. Error bars show ±1 standard error, corrected to account for the repeated-measures nature of the design [57]. There was no significant effect of stimulation condition on either accuracy or response time.

Fig 3 shows mean accuracy and response time for each stimulation condition. Accuracy and response time were analyzed using separate repeated-measures ANOVAs, with the Greenhouse-Geisser correction applied to account for non-sphericity. There was no significant effect of stimulation condition on either accuracy (*F*(1.46, 33.60) = 2.35, *p* >. 05) or response time (*F*(1.49, 34.35) = 1.04, *p* >. 05). These data contrast with the results of Marchant et al. [40], who found a robust behavioral advantage of audiovisual synchrony in a similar type of target-detection task. While no firm conclusions can be drawn from the present null result, our accuracy data actually suggest, if anything, a trend in the opposite direction, i.e., towards poorer performance in the presence of synchronous sounds. Further interrogation of the data revealed that this was due to small (not statistically significant) increases in both the mean number of missed targets and the mean number of false alarms in the AV-SYNC condition. The mean number of button presses (hits + false alarms) was consistent across the four stimulation conditions (A-ONLY: 20.9; V-ONLY: 20.9; AV-SYNC: 21.7; AV-ASYNC: 21.1; RM-ANOVA *F*(1.75, 40.15) = 0.33, *p* >. 05).

Interestingly, the pattern of accuracy scores for visual target detection (Fig 3, upper panel) mirrored that of the fNIRS beta weights (Fig 2E). That is, for both accuracy scores and beta weights, mean values were similar in the V-ONLY and AV-ASYNC conditions, but lower in the AV-SYNC condition. Based on the findings of combined fMRI–behavioral studies into the effects of audiovisual timing [39, 40, 68], it was our *a priori* expectation that behavioral performance would be positively related to the strength of the fNIRS response from visual cortex. To test for a relationship between the strength of the fNIRS response and target-detection accuracy, while accounting for individual differences in the global strength of the fNIRS response, we calculated the Pearson correlation coefficient between the magnitude of the suppressive effect of synchronous sounds on the fNIRS response (Beta_V-ONLY_ − Beta_AV-SYNC_) and any corresponding reduction in target-detection accuracy (Accuracy_V-ONLY_ − Accuracy_AV-SYNC_). A moderate, positive correlation was found (*r* = .46, *p* = .023, two-tailed). However, this correlation may have been driven primarily by a handful of participants who showed a relatively large effect in one direction or the other (Fig 2F). A robust regression analysis (MATLAB *robustfit* function, default ‘bisquare’ weighting function), which provides protection against an undue influence of potentially unrepresentative outlying data points, failed to confirm a significant relationship (*p* = .136). This null result, together with the absence of any correlation between the magnitude of fNIRS response suppression and a slowing of behavioral response time (*r* = .02, *p* >. 05), suggests that further study is needed to confirm whether there is a direct correspondence between fNIRS response strength and behavioral performance in this task.

## Discussion

To our knowledge, this is the first fNIRS study to examine multisensory interactions in ‘sensory-specific’ adult cortex. We showed that fNIRS is capable of measuring a modulatory influence of sounds on activity in visual cortex. This modulatory effect depended critically on the relative timing of auditory and visual events: only synchronous sounds modulated the visual response compared to a visual-only baseline; asynchronous sounds did not. At the group level, sounds had no significant effect on either the speed or accuracy with which participants were able to detect occasional higher-contrast visual targets.

### An unexpected suppressive effect of audiovisual synchrony

Previous fMRI studies have consistently reported stronger cortical activation to synchronous than to asynchronous trains of auditory and visual events [9, 38–40]. Across studies, this pattern has been observed in a range of cortical areas, including low-level auditory and visual cortices. Thus, the suppressive effect of synchronous sounds on visual-cortex activation (compared to both a visual-only baseline and to asynchronous audiovisual stimulation) that we observed here using fNIRS was contrary to our predictions. While sub-additive multisensory interactions (a response to bimodal stimulation that is smaller than the sum of the responses to the constituent unisensory stimuli) have often been reported in human neuroimaging studies [6, 11, 14, 47], we had not expected synchronous sounds to have a truly suppressive effect (a response to bimodal stimulation that is smaller than the dominant unisensory response). Naturally, the question arises as to whether differences between the imaging techniques used might have been critical. Previous studies that directly compared fNIRS and fMRI measurements in visual cortex have consistently found fNIRS responses to be strongly correlated with the fMRI BOLD signal, both temporally and spatially, albeit with poorer signal-to-noise ratio and a bias towards superficial cortical regions in fNIRS data [42, 66, 69, 70]. Thus, notwithstanding that our current fNIRS responses cannot be accurately localized to specific sub-areas of visual cortex, it seems unlikely that the discrepant result between the present fNIRS study and previous fMRI studies can be attributed to inherent differences between the two imaging modalities. Furthermore, contrary to previous studies [39, 40], our behavioral data suggested, if anything, a trend towards poorer performance in the presence of synchronous sounds, consistent in direction with the suppressive effect on the fNIRS response. This suggests that stimulus and/or procedural differences between studies may have been critical.

Considering stimulus differences first, it seems unlikely that our use of centrally, as opposed to peripherally, presented visual stimuli can directly account for the current findings, given that numerous other studies have demonstrated multisensory enhancement for centrally presented stimuli [38, 47–50]. However, our data could plausibly reflect an influence of stimulus salience, potentially exacerbated by the central presentation. Multisensory integration typically obeys the principle of inverse effectiveness, which was derived from single-unit studies in animals, and which states that multisensory enhancement is strongest when responses to the constituent unimodal stimuli are weak [36]. This principle has also been found to apply in human neuroimaging studies of multisensory integration [33, 50, 71] (although see [72] for some statistical considerations). There is some evidence to suggest that multisensory enhancement not only becomes weaker when the constituent unimodal stimuli are made more salient, but that it might even reverse direction and become suppressive. For instance, Noesselt et al. [33] demonstrated significant enhancement of BOLD activation when a lower-intensity visual target was paired with a co-occurring sound, but noted that, when the same sound was added to a higher-intensity visual target, any trends were if anything suppressive instead. This observation based on human fMRI data is consistent with animal electrophysiology data from Kayser et al. [73], who found that synchronous visual stimulation tends to enhance neuronal firing rates in auditory cortex when the auditory input is weak, but to suppress activity when the auditory input is more efficacious in driving neuronal responses. Thus, the suppressive impact of synchronous sounds on visual processing observed in the present study could conceivably reflect that our visual stimulus was capable of eliciting an overly strong unisensory response, especially in the superficial cortical regions (which represent the central visual field [54]) presumed to have contributed dominantly to our fNIRS measurements. Indeed, although our standard visual stimulus had a moderate contrast ratio of 15%, reversing checkerboards with contrast as low as 8% have been found to elicit an fNIRS response from visual cortex that already reaches about two-thirds the amplitude of the maximal response to high-contrast stimuli [25]. As in past fMRI and EEG studies [33, 50, 71], future fNIRS studies should consider parametrically varying stimulus salience/intensity, in order to determine how this affects the magnitude and direction of any multisensory interactions.

An emerging theme in the multisensory literature is that, instead of following a strict set of rules and principles, multisensory integration is in fact highly flexible and adaptive to stimulus context and behavioral goal (see [74] for a recent review). Although the behavioral task used in the present study was similar to that used by Marchant et al. [40], procedural differences may have contributed to the conflicting results between studies. One potentially important difference is that, in [40], targets could occur in either the auditory or visual modality with equal probability, and so participants had to continuously divide their attention between both modalities. In contrast, in the present study, targets occurred in only one modality at a time (dependent on the stimulation condition), and so, in effect, participants were encouraged to selectively attend to a single modality. It has previously been demonstrated that selectively attending to a single modality can, in some circumstances, prevent the integration of congruent multisensory stimuli [49, 75]. However, it is worth noting that the study of Lewis and Noppeney [39] also required participants to attend only to the visual modality, and yet they found synchronous, task-irrelevant sounds to enhance BOLD activation in visual and auditory areas, and to improve behavioral performance in a visual motion discrimination task. As such, differences in how attention was deployed between the auditory and visual modalities may be insufficient to fully explain the present results. Nonetheless, future fNIRS studies of multisensory processing may wish to explicitly assess the influence of selective versus divided attention across sensory modalities.

A further possibility is that the differences in fNIRS response strength between stimulation conditions might have been driven by a residual task-related systemic effect. For example, differences in behavioral performance could have led to changes in heart rate, which could in turn have affected the magnitude of the fNIRS response. To assess this possibility, we ran a series of supplementary analyses (see S1 Appendix) based on the marginal linear model [76], testing for differences in fNIRS response strength after controlling for accuracy, response time, and the total number of button presses in each condition. Significant differences between stimulation conditions remained after controlling for these behavioral measures, suggesting that the differences in fNIRS response strength are unlikely to have been mediated by changes in behavior. Interestingly, these analyses did reveal weak, yet statistically significant, overall effects of accuracy and the total number of button presses on the fNIRS response. The strength of the fNIRS response generally increased with increasing accuracy, and decreased with increasing number of button presses. However, rather than indicating causal effects of behavior on the fNIRS response, these relationships may simply reflect the influence of an unobserved common factor that affected both imaging and behavioral outcomes similarly, e.g., differences in overall attentiveness between participants.

### Cross-modal activation of visual cortex by sounds

The principal finding of the present study was of a robust modulatory effect of synchronous sounds on visually evoked activation in visual cortex. However, it is interesting to note that we also observed significant, albeit relatively weak, activation of visual cortex in the ROI to auditory stimulation alone. Unfortunately, the experimental design does not allow us to say for certain whether this reflects genuine sound-evoked activation of visual cortex, or rather a general arousal effect associated with performing a task versus resting. Functional NIRS may be particularly susceptible to such non-specific effects because of its high sensitivity to systemic hemodynamic fluctuations in the scalp [77]. Nevertheless, our study joins a collection of human neuroimaging studies that have reported positive sound-evoked activation in visual cortex, e.g., [11, 15, 78]. However, other studies have shown deactivation of visual cortex during auditory-only stimulation compared to rest [46, 79, 80], with the strength of the deactivation thought to increase with auditory task difficulty [81]. The positive activation in visual cortex in response to auditory-only stimulation observed in the present study could conceivably have been influenced by experimental context: the auditory-only blocks were interleaved with blocks in which auditory and visual stimuli were paired, which may have set up an expectancy in participants regarding an association between events in the two modalities [82, 83].

### A role for fNIRS in multisensory research?

We have shown that fNIRS is capable of measuring a modulatory influence of sounds on activity in visual cortex, suggesting that the technique may find useful application in the field of multisensory research. Specific contexts in which fNIRS might offer practical advantages over alternative neuroimaging modalities include studying the development of multisensory processing in infants [18, 20], and exploring the consequences for multisensory processing of neurological damage and/or sensory deprivation in clinical populations [84]. For instance, we are exploiting the compatibility of fNIRS with cochlear implantation [85] to study cross-modal reorganization associated with deafness and subsequent restoration of hearing [86]. The practical advantages of fNIRS may open further avenues in multisensory research, as the technique is well suited for use outside the traditional laboratory environment [87, 88] and for performing brain imaging during natural social interactions [89].

Functional NIRS is not, however, without its challenges. Obtaining reliable contact between the optodes and the scalp can be problematic on participants with thick or dark hair, although recent developments in optode design may help to mitigate this issue [90]. Even when good optode contact has been achieved, the limited depth penetration, moderate spatial resolution, and lack of an anatomical image can pose challenges in fNIRS. With current technology, fNIRS is not suitable for imaging brain areas deeper than the outermost 10–15 mm of intracranial space [91], nor for differentiating activation in cortical areas separated by less than the source–detector optode spacing, typically on the order of a few centimeters. In the context of multisensory research, then, fNIRS may be best suited to studying the hemodynamic consequences of effects that are thought to occur in a fairly widespread manner across a given sensory cortex, such as cross-modal phase reset of ongoing oscillatory activity [17, 92]. Such studies would stand to benefit from the simultaneous measurement of neuronal phase dynamics, given the relative ease with which fNIRS can be combined with EEG [93]. However, fNIRS is unlikely to be suitable for addressing research questions that require fine-grained spatial resolution, for example, in the study of distinct sub-regions of multisensory STS that preferentially respond to multimodal stimuli with specific timing relationships [94].

It is worth reiterating that fNIRS is a rapidly developing technique, with continued improvements in image quality and spatial resolution being achieved through advancements in system design [27, 95], methods for spatial registration of fNIRS data that facilitate accurate image reconstruction on anatomical brain models [96], and signal processing algorithms that specifically target the various sources of noise encountered in fNIRS recordings [97]. In the present study, we successfully took advantage of two such algorithms: a wavelet filtering approach to motion artifact correction [62], and a correlation-based approach to extracting estimates of the functional and systemic components of the hemodynamic response [65]. Repeating the analysis with these two processing stages bypassed did not alter the pattern of the results, but did result in more variable responses which, in turn, reduced the observed statistical power. The significant difference between synchronous and asynchronous audiovisual conditions, a critical finding in this experiment, no longer reached statistical significance when these two steps were omitted. The present study therefore demonstrates the practical benefit that these recently developed signal processing algorithms can provide when applied to a real fNIRS dataset.

## Conclusion

The present study demonstrated that fNIRS is capable of measuring a modulatory influence of sounds on activity in adult visual cortex. The data suggested a positive relationship between the strength of the fNIRS response and visual target-detection accuracy, although this requires confirmation in future studies. Contrary to previous fMRI studies, we found synchronous sounds to have a suppressive effect on the visual response. Given the known sensitivity of multisensory interactions to contextual factors, this discrepancy may be attributable to stimulus-related and/or procedural differences between studies, possibly related to adaptations made here to suit the fNIRS imaging modality (e.g., the use of efficacious, centrally presented visual stimuli). As the technique continues to develop, fNIRS may open new avenues in multisensory research, particularly in relation to testing in naturalistic environments and with infant and clinical populations.

## Supporting Information

S1 Appendix Supplementary analysis. (PDF)

S1 Dataset fNIRS imaging and behavioral data. (XLSX)
