# Supplementary material for: A Synchrony-Dependent Influence of Sounds on Activity in Visual Cortex Measured Using Functional Near-Infrared Spectroscopy (fNIRS)
Source: PLoS One. 2015 Mar 31;10(3):e0122862. doi: 10.1371/journal.pone.0122862 (PMC4380402; doi:10.1371/journal.pone.0122862)
Supplement: S1 Appendix — (PDF) [file pone.0122862.s001.pdf]

Wiggins, I. M. and Hartley, D. E. H., *A Synchrony-Dependent Influence of Sounds on Activity in Visual Cortex Measured Using Functional Near-Infrared Spectroscopy (fNIRS)*

S1 Appendix. Supplementary analysis

Introduction

The main analysis presented in the manuscript is based on the traditional multivariate repeated-measures analysis of variance (RM-ANOVA), applied separately to the fNIRS beta weights and the behavioral measures of accuracy and response time. Here, we present the results of an alternative analysis of the fNIRS beta weights, based on the marginal linear model. The marginal linear model, or "population-averaged" model, is similar to a linear mixed model, but without the explicit inclusion of any random effects [1]. An advantage of this alternative approach over the traditional RM-ANOVA is that it allows for the inclusion of time-varying covariates. Here, we use the marginal linear model to assess whether fNIRS response strength differed between stimulation conditions, after controlling for any differences in response time, accuracy, or the total number of button presses. The aim is to assess the extent to which differences in fNIRS response strength might have been mediated by changes in behavioral performance, perhaps through a residual task-related systemic effect.

Model specification and estimation

In the marginal linear model, stimulation condition was included as a repeated fixed effect. Since it was necessary in our original RM-ANOVA analysis to apply a correction to account for non-sphericity, we did not make any assumptions about the structure of the covariance matrix for this repeated effect, and accordingly fit an unstructured covariance matrix [1]. Additional fixed effects

were included for any continuous covariates, namely the behavioral measures of accuracy, response time, and total number of button presses. Since the models included only fixed effects, we used maximum likelihood (ML), rather than restricted maximum likelihood (REML), for model estimation (as recommended in [2]).

Example syntax for running a model that included accuracy as a covariate in SPSS Version 21 (IBM Corp, Armonk, NY) is shown below.

```
MIXED HbO_Beta BY StimCond WITH Accuracy
  /FIXED=StimCond Accuracy | SSTYPE(3)
  /METHOD=ML
  /PRINT=SOLUTION TESTCOV
  /REPEATED=StimCond | SUBJECT(Participant) COVTYPE(UN)
  /EMMEANS=TABLES(StimCond) COMPARE ADJ(BONFERRONI).
```

Results

Replication of the results of the original RM-ANOVA analysis

We first ran the marginal linear model without any covariates, to confirm that we could replicate the results of the original RM-ANOVA analysis presented in the manuscript. Indeed, this model indicated a strongly significant effect of stimulation condition on fNIRS response strength ($F(3, 24) = 22.50$, $p < .001$). After Bonferroni correction, there were significant pairwise differences between the A-ONLY condition and all other conditions (all $p < .001$), and, amongst the conditions that included visual stimulation, between AV-SYNC and V-ONLY ($p = .001$), and between AV-SYNC and AV-ASYNC ($p = .025$). These results closely mirror those of the original RM-ANOVA analysis.

Inclusion of response time as a single covariate

The inclusion as a covariate of each participant's mean response time to targets in each stimulation condition had no material impact on the model outcome. The effect of response time itself was not significant ($F(1, 46.04) = .01$, $p = .921$). Significant pairwise differences in fNIRS response strength between stimulation conditions, after controlling for mean response time, were all identical to the model without any covariates.

Inclusion of accuracy as a single covariate

The inclusion as a covariate of each participant's accuracy score in each stimulation condition revealed a significant effect of accuracy ($F(1, 38.32) = 6.88$, $p = .012$). Overall, there was a weak, positive relationship between accuracy and fNIRS response strength. However, a significant effect of stimulation condition on fNIRS response strength remained present, after controlling for accuracy ($F(3, 21.97) = 20.20$, $p < .001$). Strongly significant pairwise differences remained between the A-ONLY condition and the three other conditions (all $p < .001$ after Bonferroni correction). A highly significant difference between AV-SYNC and V-ONLY also remained present ($p = .001$). Controlling for accuracy did, however, weaken the difference in fNIRS response strength between the AV-SYNC and AV-ASYNC conditions, resulting in this comparison narrowly missing the $\alpha = .05$ significance threshold after Bonferroni correction ($p = .073$).

Inclusion of the total number of button presses as a single covariate

The inclusion as a covariate of each participant's total number of button presses in each stimulation condition revealed a significant effect of the number of button presses ($F(1, 62.30) = 4.47$, $p = .039$). Overall, there was a weak, negative relationship between the number of button presses and fNIRS response strength. Controlling for the total number of button presses did not, however, have any impact on the significant differences in fNIRS response strength between stimulation conditions, which were all identical to the model without any covariates.

Inclusion of response time, accuracy, and total number of button presses as simultaneous covariates

The inclusion of all three behavioral measures as covariates within a single model (i.e., instead of entering them into separate models one at a time) gave results consistent with the above analyses. The same trends towards increased fNIRS response strength as accuracy increased, and reduced fNIRS response strength as the total number of button presses increased, were present, though neither effect reached significance in this combined model. Significant differences in fNIRS response strength between stimulation conditions, including the Bonferroni-corrected pairwise differences between AV-SYNC and V-ONLY, and between AV-SYNC and AV-ASYNC, remained present after controlling for response time, accuracy, and the total number of button presses.

Exclusion of the A-ONLY condition

Any effects of changes in behavior on the fNIRS response strength from visual cortex could conceivably have differed between the A-ONLY condition, in which targets appeared in the auditory modality, and the V-ONLY, AV-SYNC, and AV-ASYNC conditions, in which targets appeared in the visual modality. We therefore repeated all of the above analyses, but including only the three conditions that involved the detection of visual targets. Results were highly consistent with the preceding analyses that included all four stimulation conditions, i.e., overall, fNIRS response strength was positively related to accuracy and negatively related to the total number of button presses, but significant differences in fNIRS response strength between the V-ONLY, AV-SYNC, and AV-ASYNC conditions remained present after controlling for the behavioral measures.

Discussion

This re-analysis of the data using the marginal linear model approach indicates that the reported differences in fNIRS response strength between the four stimulation conditions remained significant after controlling for the behavioral measures of response time, accuracy, and the total number of buttons presses (in all cases except for the AV-SYNC vs. AV-ASYNC comparison after controlling for accuracy, which remained close to significance). As such, it seems unlikely that differences in fNIRS response strength were mediated by differences in behavioral performance.

These analyses additionally revealed significant effects of both accuracy and the total number of button presses on fNIRS response strength. Overall, fNIRS response strength increased with increasing accuracy, and decreased with increasing number of button presses. While interesting, it must be noted that these relationships do not necessarily imply a causal effect in either direction. They may reflect the influence of an unobserved common factor, e.g., some participants may simply have been less attentive to the stimuli than others, which would be expected to lead to generally weaker cortical responses, as well as poorer task performance (potentially manifesting as both an increase in the number of button presses and reduced accuracy).

References

1. West BT, Welch KB and Galecki AT (2006) Linear Mixed Models: A Practical Guide Using Statistical Software. Boca Raton, FL: CRC Press.

2. Field A (2009) Discovering Statistics Using SPSS. London: SAGE Publications.
